# C3-IoC: A Career Guidance System for Assessing Student Skills using Machine Learning and Network Visualisation

**DOI:** 10.1007/s40593-022-00317-y

**Published:** 2022-12-01

**Authors:** Adán José-García, Alison Sneyd, Ana Melro, Anaïs Ollagnier, Georgina Tarling, Haiyang Zhang, Mark Stevenson, Richard Everson, Rudy Arthur

**Affiliations:** 1 Department of Computer Science, University of Exeter, Exeter, UK; 2 Department of Computer Science, The University of Sheffield, Sheffield, UK; 3 Graduate School of Education, University of Exeter, Exeter, UK

**Keywords:** Career guidance system, IT sector, Technical and non-technical skills, Job network visualisation, Text mining, Machine learning

## Abstract

Artificial Intelligence in Education (AIED) has witnessed significant growth over the last twenty-five years, providing a wide range of technologies to support academic, institutional, and administrative services. More recently, AIED applications have been developed to prepare students for the workforce, providing career guidance services for higher education. However, this remains challenging, especially concerning the rapidly changing labour market in the IT sector. In this paper, we introduce an AI-based solution named C3-IoC (https://c3-ioc.co.uk), which intends to help students explore career paths in IT according to their level of education, skills and prior experience. The C3-IoC presents a novel similarity metric method for relating existing job roles to a range of technical and non-technical skills. This also allows the visualisation of a job role network, placing the student within communities of job roles. Using a unique knowledge base, user skill profiling, job role matching, and visualisation modules, the C3-IoC supports students in self-evaluating their skills and understanding how they relate to emerging IT jobs.

**Supplementary Information:**

The online version contains supplementary material available at 10.1007/s40593-022-00317-y.

## Introduction

The mismatch between new graduates and the Information Technology (IT) job market is a subject of global concern. The demand for digitally skilled workers has been on the European Digital Agenda for over a decade (European Commission, 2010). In the U.S., for instance, nearly two-thirds of the 13 million new jobs created since 2010 “required medium or advanced levels of digital skills” (Engler et al., 2018, p. 12); and in the UK, a 2016 report concluded that “a shortage in suitable digital skills for digital jobs persists in the UK labour market” (ECORYS, 2016, p. 75). However, having just the technical skills to enter the job market has proved to be insufficient (Aasheim et al., 2009; Lee et al., 1995; Ponce del Castillo, 2018; Sahin & Celikkan, 2020). Furthermore, several studies reveal the need for a balance of technical and non-technical skills in the IT field (Bailey, 2014; Sahin & Celikkan, 2020) and emphasising the lack of personal, or so-called ‘soft skills’ (Matteson et al., 2016), among IT graduates, such as communication, teamwork, analytical thinking, creativity, and leadership (Sahin & Celikkan, 2020). In addition, recent reports reveal high unemployment rates amongst Computer Science graduates (Shadbolt, 2016). Therefore, there is a pressing need to help students better understand which skills are relevant for emerging jobs and develop self-awareness about their skillset, both technical and non-technical.

One way of personalising student support for career exploration is through expert systems—also known as computer-assisted career guidance systems (CACGS) – which have been developed to help users stay abreast of the latest job and skill requirements (Ashok et al., 2018; Mujtaba & Mahapatra, 2019; Oentaryo et al., 2018). However, few online systems encompass technical and non-technical skills in the IT sector, and even fewer place students on a career path accordingly. This paper presents an online career-guidance system named C3-IoC,^1^ which helps undergraduates and high school students explore job roles in the IT sector and transition from education to employment. The novelty of this AI-based solution is in using machine learning and text mining techniques that accurately relate a range of skills to existing IT jobs. The system consists of three modules: (1) a novel *knowledge base* covering the full range of skills, both technical and non-technical, that are necessary for emerging jobs in the IT sector; (2) a *user skill profiling module* in which students’ technical skills are collected using a CV parser and non-technical skills are self-assessed through a questionnaire; and (3) a *job role matching and visualisation module* where the collected skills are used to project users onto a network of matched job roles. This solution provides students with both a personalised visualisation of their career field and an effective way to consider and plan IT career options in the “job space”. In addition, by drawing on technical and non-technical skills, the C3-IoC system helps students to raise skill awareness. To validate the quality of the C3-IoC system, we conducted a comprehensive evaluation of the accuracy of the job role matching module using “dummy” data and a user trial evaluation of student experience with the platform.

## Related Work

Career guidance systems build on a history of work in the field of vocational guidance dating back to the 1970s, with tools such as the System of Interactive Guidance Information (SIGI) (Katz, 1973) and the DISCOVER (Rayman & Harris-Bowlsbey, 1977). Studies have demonstrated the benefits of these systems in student self-assessment and career exploration (Fowkes & McWhirter, 2007), such as ease of search within large amounts of occupational and educational information, awareness of interests and abilities, and the ability to make informed decisions (Betz & Borgen, 2010; Gati, 1994; Neault & Saunders, 2012). They usually include career assessment, with automated scoring and searchable databases describing occupations, colleges and academic disciplines (Gore & Hitch, 2005). As recent examples, González-Eras and Aguilar (2019) proposed a model to align academic profiles and job advertisements based on student competencies, and Nguyen et al. (2018) developed a decision support system to assist students in identifying positions most related to their interests.

However, these assessments cannot be reduced to a matching process between an individual’s technical skillset and job role (Majid et al., 2012; Metz & Jones, 2013; Ngang Tang, 2019). The importance of including non-technical skills, interests, values, aptitude, and personality traits is essential to assess individuals in making informed decisions regarding their careers. The diversity of online career systems offers students a wide range of opportunities to explore their skillset and understanding of careers. Some systems are based on self-assessment of skills and values (e.g. Skill Matcher^2^), while others combine technical skills with personal interests, aptitudes, and personality traits (e.g. FOCUS-2,^3^ O*NET Interest Profiler,^4^ Morrisby,^5^ Prospect^6^). Popular career systems such as Myers-Briggs Type Indicator^7^ (MBTI), Strong Interest Inventory,^8^ Careerscope,^9^ FOCUS-2 and O*NET Interest Profiler were designed to be applied in virtually any market. Other systems, such as the CAPA Integrative Online System for College Major Exploration, provide suggestions of college major clusters based on individuals’ patterns of interest and confidence levels.

In order to provide accurate information, career guidance systems rely on searchable databases. The most comprehensive source of free occupational information is the U.S. Department of Labor’s O*NET (Peterson et al., 1999), used in online platforms such as CareerScope, My Next Move and CareerOneStop.^10^ The O*NET database contains hundreds of standardised and occupation-specific descriptors continually updated by surveying a broad range of workers. In total, O*NET gathers information about 969 occupations from the Standard Occupational Classification (SOC) system, using 277 descriptors. In Europe, another relevant public taxonomy is ESCO (European Skills, Competences, Qualifications and Occupations) (De Smedt et al., 2015). The ESCO classification identifies and categorises skills, competencies, qualifications and occupations relevant to the EU labour market and education field. In total, ESCO gathers information about 5380 occupations, 5737 skills and 20 qualifications.

Whilst ESCO and O*NET have widely used databases in career systems, and both have limitations when addressing the specific needs of the IT sector in the UK labour market. For example, they miss relevant up-to-date skills and roles because they are research-based instead of emerging directly from live labour market changes (e.g. job ads). A recent report highlights that the US O*NET database lacks details regarding the IT job sector, with typical roles such as ‘data scientist’ not being mentioned (Hillage & Cross, 2015). Also, ESCO does not consider discrete levels of importance of the skills required for specific job roles. As far as we know, there is no standard knowledge base ontology that covers and weights technical and non-technical skills by combining research-based taxonomies and data emerging from the current IT labour market. In the C3-IoC system, we therefore use two complementary datasets to create the C3-IoC knowledge-base, namely: (1) a corpus of IT job advertisements curated from the Department of Work and Pensions “Find a job”^11^ website from which both technical and non-technical skills from relevant jobs were extracted; and (2) a corpus of general jobs taken from the O*NET^12^ taxonomy from which we drew our knowledge of the technical and non-technical skill requirement of different job roles.

## The C3-IoC System

### Motivation for the C3-IoC System

In the field of AI and career guidance systems, three important aspects of current provision can be improved (De Smedt et al., 2015; Hillage & Cross, 2015; Peterson et al., 1999). Firstly, working with large datasets to create repositories of jobs and skills which are fine-tuned to specific sectors is essential to keep up-to-date with emerging and changing roles. The IT sector is a prime example where this is highly pertinent. Secondly, automating the process of skill profiling makes it less time-consuming for the user and improves understanding of skills and job roles. Lastly, providing visualisations and advanced metrics to accurately guide and place users in the job space. Given the skill gap between education and industries, especially in the IT sector, the development of the C3-IoC system was motivated by a desire to explore these three opportunities and to produce an online career guidance system for students that would give them greater awareness of their skillset and of the skills required to pursue certain roles in IT.

**Fig. 1:**
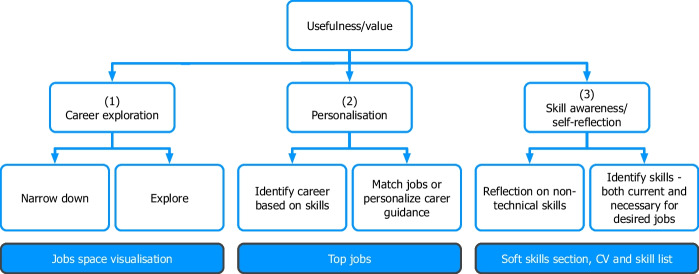
Perceived value of the pilot version according to students

In developing this iteration of the C3-IoC system, we improved upon a previous pilot version, drawing on findings from student perceptions of the platform. The pilot version was essential to understand the usefulness for students in using a system with the aspects mentioned. The pilot consisted of a CV parser, a non-technical skills questionnaire and a network visualisation of the IT job space. This first version's matching process was limited to technical skills and IT job roles. We then conducted a mixed-method user trial with 71 participants using surveys and interviews. The findings from the pilot showed that students found the system useful along three main dimensions: career exploration, personalisation and skill awareness (see Fig. 1).

Firstly, *career exploration* was regarded as the most valuable aspect of the pilot system. Students perceived it as helpful in giving a broad overview of the job market, especially those who had no idea what roles to pursue. Additionally, the job role visualisation was considered more valuable over an internet search. Secondly, students appreciated a personalised career coaching experience which would guide them to job roles based on their *personal skillset*. They also liked being told what skills they needed to develop for certain job roles and seeing how closely their skillset matched those. Finally, in terms of *skill awareness* and *self-reflection*, students found the system useful in helping them think about their skillset and what they display in their CVs. It particularly helped them reflect on the relevance of non-technical skills in the workplace. However, although the pilot version allowed for skill awareness through a self-assessment questionnaire, it did not present a profile of non-technical skills nor positioned the user in the job space accordingly. Informed by the potentialities of the pilot version, the final version of the C3-IoC sought to address these aspects by providing improved visual feedback and relating student technical and non-technical skills with job roles.

### The C3-IoC System Architecture

Building on our pilot study, the overall aims of the current C3-IoC system are to provide a personalised career exploration system and help users understand the key technical and non-technical skills required for careers in the IT sector. Although the system primarily focuses on the IT sector, it also incorporates information about a wide range of job roles at a coarse resolution to allow users who might consider themselves outside the IT sector to explore potential roles.

**Fig. 2:**
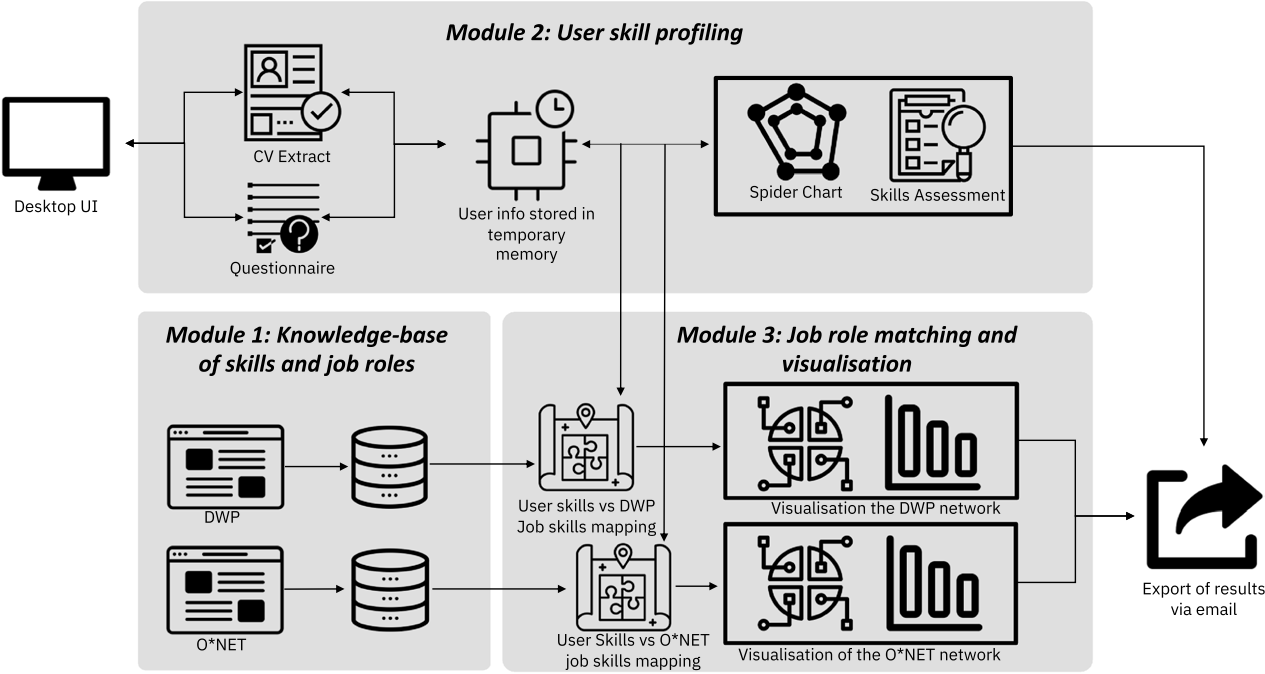
C3-IoC system architecture comprised of three main modules

**Fig. 3:**
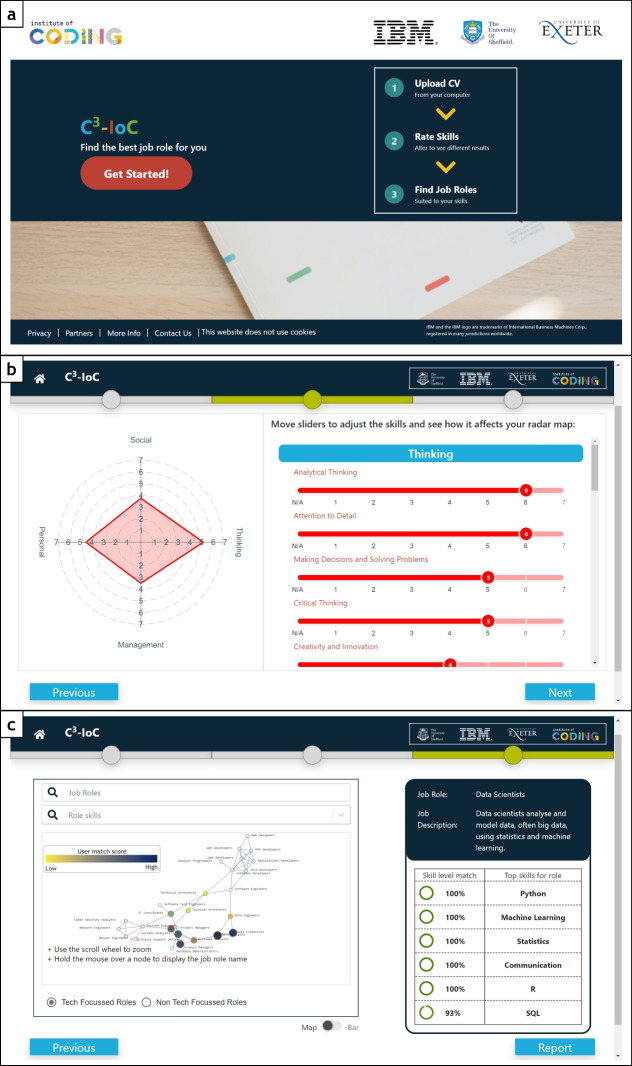
C3-IoC screenshots: welcome screen (**a**), radar chart of non-technical skills ratings (**b**) and network map of a user in the job role space (**c**)

The system architecture underlying the C3-IoC website consists of three main modules: (1) a knowledge base of skills and job roles; (2) a user skill profiling module; and (3) a personalised job role matching and visualisation module, which is shown in Fig. 2. A typical user journey through the C3-IoC system is illustrated in Fig. 3. Users first encounter a welcome screen (a) which offers an overview of the system. They then go through a three-stage journey, with progress marked via a progress bar at the top of the screen. The first stage extracts user skills information via a CV parser and a questionnaire. The second stage allows the user to refine their skill profile. For example, the middle screenshot (b) illustrates a radar chart of non-technical skills whose levels the user can adjust. Finally, the third stage of the system allows users to explore job roles: the bottom screenshot (c) shows a network visualisation of the user’s placement in the IT job role space.

#### Module 1: Knowledge-base of Skills and Job Roles

The first gap in the current provision that we wanted to address with this website was the lack of a comprehensive landscape of the technical and non-technical skills required for existing and emerging jobs in the IT market. To achieve this, we used classical techniques from Natural Language Processing to process and extract information from job ads (Jurafsky & Martin, 2008). The data was scraped came from two sources, for reasons described below, and we then merged the findings from both to create a non-technical skills list and questionnaire.

##### Identification of Job Roles and Skills from the IT Job Corpus

The first step in this process was to curate a corpus of recent job vacancies in the UK IT sector from the “Find a job” website. This website hosts job advertisements classified into 29 categories, including IT. The website was crawled weekly from October 2018 to December 2019, and all jobs in the IT category were extracted. Collected job metadata, including the job title, job description and URL. Duplicated jobs were removed, resulting in a corpus of 22,359 jobs. We will refer to this dataset as the *IT job corpus.*

A comprehensive list of technical skills was then extracted using text mining techniques from the corpus. A predefined list of 100 common technical skills (e.g. “Python”, “Agile”) was used as a seed list to extract a complete list of skills. This was achieved by training a Word2Vec model (Mikolov et al., 2013) on the corpus to represent words as latent vectors. The ten most similar words to every word in the seed list were found using cosine similarity and extracted. These words were combined with the original seed list terms to generate an extended skill list consisting of 902 terms. However, the extended skill list was noisy and included some non-skill words and synonyms. We manually removed the noise terms and merged synonyms, resulting in an IT skill dictionary consisting of 195 unique skills. A manual examination of the skills revealed they could be classified into four categories: general tech, programming languages, tools and platforms, and non-technical skills, including specific training (see Table 1 for category sizes and examples). In our final knowledge base, the non-technical skills were made a separate dimension, keeping only the specific training (e.g. machine learning) as part of the IT skills, as described further in the section about the second module.

**Table 1:** *IT job corpus* skill categories: number of skills (N. Skills) and examples

Skill Category	N. Skills	Skill examples
General Tech	85	Agile, UX, Web design
Programming Languages	20	Python, C#, Java
Tools and Platforms	63	Azure, Cisco, Excel
Non-technical	25	Communication skills, statistics, machine learning

The task of clustering IT job advertisements into common job roles (such as “software engineer” or “data scientist”) is non-trivial. There are no standard definitions of these roles, and the same role may have multiple names and not all job advertisements necessarily fall into one of the roles. We identified common job roles by analysing the job title data. This data was frequently noisy, including, for example, locations or salaries. The titles were first cleaned by removing all numbers and punctuation, in addition to common locations and role level signifiers (such as “graduate”). All cleaned titles which occurred more than 100 times were then extracted and manually examined to produce a list of 26 job roles. Job advertisements matching one of these roles (11,784) were assigned to that role, while those that did not match were discarded. The skills occurring in each remaining job advertisement were then extracted using the skills list described above. Finally, each job role was represented as a weighted list of skills, where the weighting of a skill is given by the proportion of ads in that role containing that skill. Table 2 shows the 26 extracted job roles, the number of jobs each role contains and its most common skills.

**Table 2:** The 26 job roles extracted from the *IT job corpus*, along with the frequency of jobs and the most common skills for that role

Job role	Freq	Top three skills
Software developer	2374	SQL, communication skills, Agile
Software engineer	1479	Agile, communication skills, Java
IT technical support officer	822	computer hardware, communication skills, troubleshooting
Web developer	767	JavaScript, CSS, HTML
Network engineer	767	communication skills, Cisco, computer hardware
Data analyst	542	Excel, communication skills, SQL
Business analyst	487	communication skills, Agile, business analysis
Systems engineer	483	communication skills, VMWare, Windows Server
IT project manager	413	project management, communication skills, Agile
Solution architect	403	communication skills, Agile, Amazon Web Services
Java developer	320	Java, Agile, SQL
Applications developer	303	communication skills, Agile, Java
Data engineer	296	Python, SQL, Agile
Data scientist	281	Python, machine learning, statistics
Devops engineer	256	devops, Amazon Web Services, Linux
Web designer	211	UX, HTML, CSS
Database administrator	201	SQL, database administration, Oracle
Product manager	175	communication skills, Agile, innovation
Software test engineer	175	automated testing, Agile, test scripts
Cyber security analyst	171	cyber security, information security, communication skills
IT consultant	164	communication skills, SQL, Agile
.net developer	154	.Net, C#, SQL
Php developer	150	PHP, JavaScript, CSS
Systems analyst	140	communication skills, SQL, Excel
Technical architect	140	communication skills, Agile, Java
Analyst programmer	110	SQL, communication skills, C#

##### Identification of Job Roles and Non-technical Skills from the O*NET Database

To complement the collection of IT jobs, we drew on a second data set so that users of the system who were not currently in IT could orient themselves. This data set would allow us to provide a visualisation of how ‘far’ from their desired job in the IT sector a user was. The O*NET program conducted by the U.S. Department of Labor produces the publicly available O*NET database detailing the importance of 231 workplace skills, knowledge, and abilities for the completion of each of the 967 occupations (hereinafter referred to as job roles) recognised under the Standard Occupational Classification (SOC) System. The O*NET database is updated annually, allowing for snapshots of the relationships between job roles and skills through a continual survey of workers from each occupation. We used the most recent annual O*NET database corresponding to the year 2019 (version db_24).

A second important benefit was that the O*NET data set offered a much more nuanced overview of the non-technical skills required in a range of jobs. As these are often transversal skills, it can be challenging to determine their weighting in different job roles. O*NET’s extensive research background in matching skill levels and job roles based on interviews and surveys with employees provides a rich understanding of, for instance, how important non-technical skills like ‘attention to detail’ are for different job types.

A final benefit was that O*NET also offers a comprehensive set of questions for users to self-evaluate against these skills. This latter was important because assessing non-technical skills via questionnaires comes with limitations. On the one hand, for example, it is complex to address with only one or two questions whether someone knows how to communicate effectively in multiple forms (e.g., orally, written, visually). On the other hand, self-assessment is subjective. For this reason, it was important to have a tested and reliable survey.

From the O*NET database, we created a matrix in which each row corresponds to one of the 967 listed job roles and each column to one of the 231 skills measured by O*NET. Using the *Education, Training and Experience* table in O*NET, we counted the percentage of respondents who ranked the necessary qualification for each job role at level 6 (Bachelor’s Degree) or higher. We retained only job roles; over 50% of the respondents believed the job role required a level 6 or higher qualification. We did this to focus our efforts on users who are university students or recent graduates. This restriction removed a large number of job roles from consideration, most of which could be described as ‘physical’ or ‘manual’ (Alabdulkareem et al., 2018).

O*NET estimates the importance of each attribute or skill for every job role. We therefore represented job roles as vectors of skill weightings. To measure the attributes which are relevant for jobs requiring a degree, we correlated the vector, $d\left(j\right)$, which measures the proportion of respondents who ranked the necessary qualification or skill for each job role, $j$, as level 6 or higher with the vector $v$, where $v\left(j\right)$ is the importance of attribute $s$ for occupation $j$. If the correlation was greater than 0, we retained that skill. As a result, in this system iteration, we considered a total of 142 skills and 381 job roles from the O*NET database. We will refer to this as the *general job corpus* for the remainder of this paper.

##### Merging of Non-technical Skills from Both Data Sets

As stated above, the decision was taken to draw on O*NET questionnaires for their reliability and skill level discrimination. Since the C3-IoC uses two data sets – the *IT job corpus* and the *general job corpus –* a matching between both was therefore needed so that users’ responses to the non-technical skills questionnaire could be directed to both data sets.

To identify users’ non-technical skills, we selected a set of questions from four O*NET questionnaires: *Skills* (S), *Abilities* (A), *Work Activities* (WA) and *Work Styles* (WS). The starting point was the list of 25 non-technical skills from the *IT job corpus*, which were then matched with the 144 questions from O*NET. The matching between both lists followed an extensive manual review with several rounds of peer revision. The selection criteria followed two main questions: (1) which O*NET questions best describe the *IT job corpus* non-technical skills? (2) which non-technical skills are relevant for IT jobs based on previous findings and literature? During this process, we could not find matches for five *IT job corpus* skills, as displayed in Table 3, so these were not included in the final list used for the C3-IoC. Additionally, three extra skills from O*NET were included in accordance with our second criterion. For certain skills, there was a need to have more than one question to describe that skill better. The questionnaire was then presented on the website using O*NET questions, scale and examples. For instance, for “creativity and innovation”, the question *How confident are you in thinking creatively*? was asked, together with a scale from 1 to 7 with examples in categories (1) “change the spacing on a printed report”, (4) “adapt popular music for a high school band”, and (6) “create new computer software”. Four categories were created for the final 24 questions regarding non-technical skills: thinking/cognitive, social, personal and management. Table 3 displays the final matching between both data sets. The numbers followed by the O*NET acronyms refer to the question number of the corresponding questionnaire.

**Table 3:** Matching of non-technical skills between *IT job corpus* and *general job corpus* (referring to specific O*NET questions)

Category	Non-technical skill	IT job corpus skill	O*NET questions
Thinking/Cognitive	Analytical thinking	Analytical thinking	WA9 analysing data or information
Thinking/Cognitive	Creativity and innovation	Creativity/innovation	WA11 thinking creatively
Thinking/Cognitive	Critical thinking	Critical thinking	S8 active learning
Thinking/Cognitive	Problem solving and decision making	N/A	S17 complex problem solving
Thinking/Cognitive	Mathematical Reasoning	Numeracy	A12 mathematical reasoning
Thinking/Cognitive	Attention to detail	Attention to detail	A16 flexibility of closure
Social	Communication	Communication/presentation	A4 written expression
Social	Communication	Communication/presentation	S4 speaking
Social	Communication	Communication/presentation	WA25 interpreting information for others
Social	Interpersonal	Interpersonal	WA28 interpersonal relationships
Social	Negotiation	Negotiation	S14 negotiation
Social	Teamwork	Teamwork	S12 coordination
Personal	Adaptability	N/A	WA12 updating and using relevant knowledge
Personal	Accountability	GDPR	WS13 integrity
Personal	Initiative	Initiative	WS3 initiative
Personal	Self-control	N/A	WS8 self-control
Personal	Self-motivation	Self-motivation	WS1 achievement/effort
Personal	Autonomy	Working independently	WS14 independence
Management	Leadership	Leadership	WS4 leadership
Management	Teaching and guidance	N/A	WA38 providing consultation and advice
Management	Teaching and guidance	N/A	WA35 training and teaching
Management	Administrative	Organisational/prioritise	WA15 organising, planning and prioritising work
Management	Management	Project management	WA33 coordinating work and activities
Management	Time management	Time management	S32 time management

#### Module 2: User Skill Profiling

The second challenge the system seeks to address is collecting a comprehensive overview of user skills without requiring too much user input. For users to complete their technical and non-technical skills profile (also shown as ‘Personal skills’ on the website) profile without much effort, the C3-IoC provides an automated CV scraper, skill predictions, and recommendations.

Users can upload their CVs in PDF format and a list of technical skills is extracted from it. In detail, the input file is converted to plain text then matching is performed using a predefined dictionary of technical skills derived from the *IT job corpus*. For the non-technical skills, users need to fill out a questionnaire with 24 questions that were selected based on their relevance to the IT sector, as described in the previous section. In order to fulfil the requirements for the matching process, particularly with regard to the *general job corpus,* only four of the 24 questions are mandatory, and these are displayed at the top of the questionnaire. These four questions concerning four non-technical skills are asked in a certain order of relevance and represent the *most informative* questions, which means that just by gathering answers to these questions, we can gain enough information to place the user in the job network accurately. By using the *most informative order* method, we were able to determine that the four most informative non-technical skill questions correspond to: “attention to detail”, “management”, “self-control”, and “teaching and guidance” (*training and teaching*). This method allows us to extract as much information as possible in a minimum amount of questions. To find out *the most informative order,* we defined a loss function as the (Frobenius) norm of the difference between the data matrix $X$, whose elements ${X}_{js}$ are the importance that O*NET estimates of the job $j$ and the skill $s$, and an estimate of $X$, $\overline{X }$ that is constructed from the answers to the non-technical skill questions. This estimate was constructed using the non-technical skill questions as independent variables in a regression model. That is, we solved the linear regression ${X}_{s}={X}_{Q}\beta$, where ${X}_{s}$ is the column of $X$ corresponding to skill $s$, ${X}_{Q}$ is the slice of $\overline{X }$ corresponding to the answered non-technical skill questions, $\beta$ are the unknown coefficients. We started with just one question and found the most informative question from the set, as the one resulting in the smallest loss. This turned out to be the question asking about “attention to detail”. We then took the question set found for the most informative question and found three more additional questions that would reduce the loss function the most: “management”, “self-control”, and “teaching and guidance”. As a result, we covered a broad range of work activities with four questions.

Next, after uploading their CV and filling out the questionnaire, the user is shown a list of their inferred skills, from which they can remove individual items that seem inappropriate for their profile. They are additionally prompted to consider recommended skills in the knowledge base, which they can add to their profile. This encourages the user to reflect on the skills they have developed so far and also those they have included on their CV. Once the user completes the skill list, they proceed to a page where they can visualise both their technical and non-technical skills through radar diagrams that show the rating of skills from 1 to 7, divided into four main areas. For technical skills, the four main areas are *programming languages*, *general tech*, *specific training*, and *tools and platforms*. The four domains for non-technical skills are *social*, *thinking*, *personal* and *management* skills (as shown in Fig. 2). With this interactive interface, users can refine their skills once more and watch the respective changes happening on the radar charts. This way of visualising the skills gives users an overview of the dimensions in which they have more expertise and those they need development.

#### Module 3: Job Role Matching and Visualisation

The final challenge the C3-IoC system seeks to address is how to make an accurate projection of the user into the job role network. This involves three phases: job role matching, construction of the jobs network visualisation and projection of the user into the latter. In this section, we address those in detail.

##### Job Role Matching

Both the profile and job roles are represented as weighted skill vectors. A common way to measure relatedness is to use cosine similarity, which measures the cosine of the angle between two vectors. However, this does not work in our case because we want to consider vector magnitude (corresponding to skill level). We therefore used the following metric which takes into account skill level. Let $S$ be the fixed set of skills, $S=\left\{{s}_{1},{s}_{2},\dots ,{s}_{N}\right\}$, where $N$ is the total number of skills. Each job role $j$ and user $u$ can be represented by a set of skill weightings, $j=\left\{{j}_{s}|s\in S\right\}$, $u=\left\{{u}_{s}|s\in S\right\}$. Then, the distance between $u$ and $j$ is defined as:

1 $$D\left(u,j\right)=\sqrt{\sum_{s\in S}{\mathrm{max}({j}_{s}-{u}_{s},0)}^{2}}$$

Note that the distance between $u$ and $j$ does not increase if the user has at least the requisite skill level for the job role. Therefore, the similarity between $u$ and $j$ is defined as:

2 $$Sim\left(u,j\right)=\frac{D\left(\mathrm{\varnothing },j\right)-D(u,j)}{D(\mathrm{\varnothing },j)}=1-\frac{D(u,j)}{D(\mathrm{\varnothing },j)} ,$$

where $\mathrm{\varnothing }$ denotes the empty set and the value bound of $Sim(u,j)$ is $[\mathrm{0,1}]$, such that values tending towards unity indicate a better job role matching as they indicate a better correspondence between the student profile and a job role.

##### Job Role Visualisation

Two main techniques were used in order to allow the visualisation of the job role landscape as networks into which users can be projected: (1) the *revealed comparative advantage* index (RCA), which denotes the relative importance of a skill for a job role after accounting for skills which are common across many job roles. This allows students to more effectively compare their skillset to occupational requirements (Alabdulkareem et al., 2018); (2) a force-directed layout that effectively visualises related career fields, leading students to explore skills and practices associated with a specific job or occupation. Two job role networks were displayed based on the two knowledge databases: a tech-focused (from the *IT job corpus*) and a non-tech focused (from the *general job corpus*).

To create these job role networks, we denote by $net(j, s)$ the level of importance of skill $s \in S$ to job role $j \in J$ where $J$ is the set of job roles and $net\left(j, s\right)$ indicates one of the studied databases: *IT job corpus* or *general job corpus*. Also, $net\left(j, s\right)=5$ indicates that $s$ is essential to $j$, while $net\left(j, s\right)=0$ indicates that workers of job role $j$ need not possess or perform skill $s$. This importance level or skill level rating is associated with the O*NET occupation and for simplicity, both databases were normalised in the range $[\mathrm{0,5}]$. Usually, job roles are the units of interest in labour dynamics, and they are identified by a set of skills possessed by workers of that occupation. Therefore, we need to quantify how useful a skill is for identifying a job role. RCA normalisation compares the relative importance of a skill in a job role to the expected importance of the skill on average. It indicates that an occupation relies on the skill more than expected. For example, most jobs require basic reading comprehension, so this skill is not discriminative, and the RCA transformation accounts for these effects. RCA is defined by:

3 $${\text{rca}}\left(j,s\right)=\frac{net\left(j,s\right)/{\sum }_{{s}^{\mathrm{^{\prime}}}\in S}net\left(j,{s}^{\mathrm{^{\prime}}}\right)}{{\sum }_{{j}^{\mathrm{^{\prime}}}\in J}net\left({j}^{\mathrm{^{\prime}}},s\right)/{\sum }_{{j}^{\mathrm{^{\prime}}}\in J,{s}^{\mathrm{^{\prime}}}\in S}net\left({j}^{\mathrm{^{\prime}}},{s}^{\mathrm{^{\prime}}}\right)}$$

We denote an *effective need* for skill $s$ in job $j$ by $e(j, s)=1$ if ${\text{rca}}\left(j,s\right)>1$, and $e(j, s)=0$ otherwise. We use the following network projection onto the *space of job roles* to create a similarity measure between two job roles:

4 $${J}\left(j,j\mathrm{^{\prime}}\right)=\frac{{\sum }_{s\in S}e(j,s)\cdot e\left(j\mathrm{^{\prime}},s\right)}{\mathrm{max}({\sum }_{s\in S}e\left(j,s\right),{\sum }_{s\in S}e\left(j\mathrm{^{\prime}},s\right))}$$

**Fig. 4:**
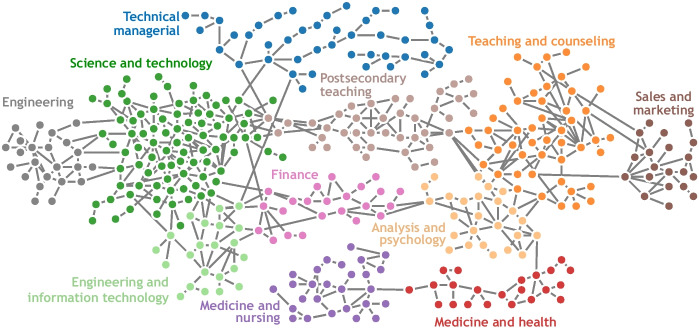
The job role landscape generated based on the *general job corpus* has 11 communities. Each node colouring corresponds to a community (manually labelled). Individual nodes refer to job roles and are linked by their skill similarity

**Fig. 5:**
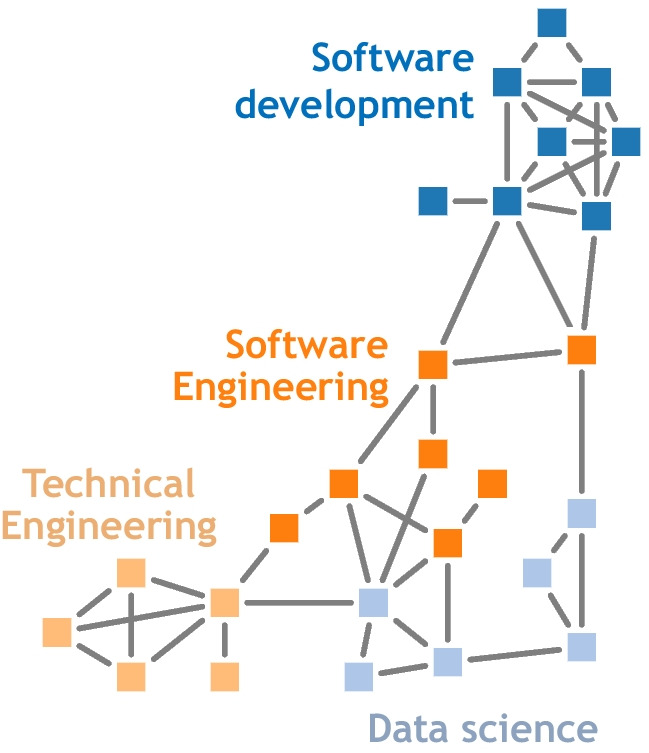
The job role landscape generated for the *IT Job corpus* database has 4 communities. Each node colouring corresponds to a community (manually labelled). Individual nodes refer to job roles and are linked by their skill similarity

This projection identifies job role pairs that share key occupational features. Defining the similarity between job roles in this way allows the “job role space” to be visualised as a network (graph) in which individual job roles are nodes or vertices and similar job roles are linked together. Ideally, the aggregate structure in the job roles network should correspond to meaningful labour dynamics. To this end, we identify job role types using the *Louvain community detection algorithm* (Blondel et al., 2008). This method identifies node communities by comparing the density of connections within a community to connections between communities. This method requires no assumptions about the number of communities to be found. Figures 4 and 5 illustrate the job roles network for the *general job corpus* and *IT job corpus* databases, respectively. In the figures, each filled circle or rectangle represents a node (job role) and the grey lines indicate the relationship between the different job roles, such that the shorter the line, the greater the similarity between the job roles. The absence of a line/edge between nodes indicates that job roles have no skills with effective needs in common.

The final feature of this module is the projection of the user onto the job role networks. Thus, considering the created job role networks and the proposed similarity measure for the job role matching, we ranked the jobs based on their similarity score to a given user (Eq. (2)).

**Fig. 6:**
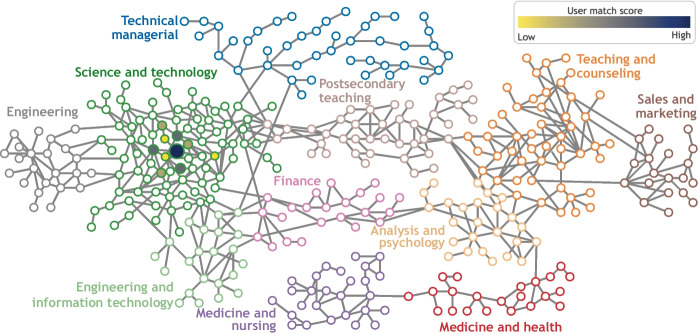
Projection of a *Bioinformatician* user profile in the non-tech focused network. The top 10 positions are highlighted using filled circles, such that the darker the colour, the better the job matching between the user skills and the job. Some of the top job role matches and scores are: *Bioinformatic Scientist (96%), Molecular and Cellular Biologist (91%), Computer Network Architect (89%), Data Warehousing Specialist (85%)*

As an example, Fig. 6 illustrates the resulting projection of a Bioinformatician user profile onto the non-tech focused network based on their input skill profile. The top 10 positions are highlighted using different levels of colour intensities, such that the darker the colour, the better the job matching between the user skills and the job roles in the *general job corpus*. These top 10 job roles are also displayed to the user as a bar chart on the website. The figure shows that the user is located in the Science and Technology community based on the input skills, which is the most suitable for the given user profile. Also, notice that all the remaining predicted job roles are in the same cluster and close to each other, which supports the robustness of the job matching component of the proposed C3-IoC system.

## Accuracy of Matching between User Profiles and Job Roles

**Fig. 7:**
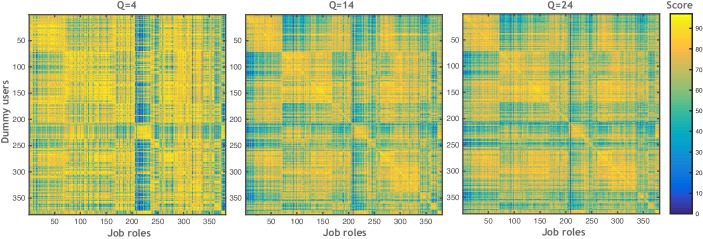
Similarity score matrices on three settings of a number of questions $Q=4$, $Q=12$, and $Q=24$

**Fig. 8:**
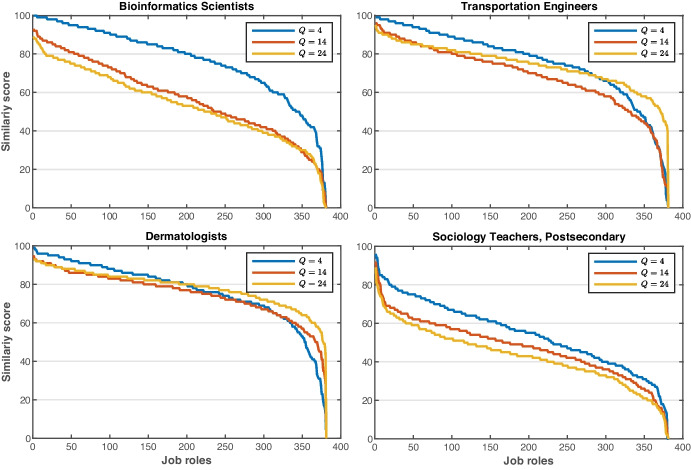
Similarity scores of four dummy users for three settings of the number of questions answered, $Q=4$, $Q=12$, and $Q=24$). The job roles are sorted in descending order according to their similarity score

In this section, we provide some validation of the accuracy of the job role matching module. Using the job profiles given in O*NET to construct ‘dummy’ users who match certain job profiles, we explore the effect restricting the number of skills has on matching accuracy. To do this, we create a dummy user with skills matching a given job role. However, the user profile is constructed assuming they had answered only $Q$ questions. A dummy user ${u}_{j}^{Q}$ was constructed for each of the $\left|J\right|$ job roles $j\in J$ and then the similarity $Sim\left({u}_{j}^{Q},j^{\prime}\right)$ was computed between the dummy users and each job role profiles $j^{\prime}\in J$. Figure 7 shows the similarity score matrices of three dummy user configurations when answering $Q=4$, $Q=14$, and $Q=24$ questions. As can be seen from the figure, the similarities become more highly differentiated as the user answers more questions and more accurate information becomes available. It is also clear that in most cases, the most similar job role to ${u}_{j}^{Q}$ is $j$ as shown by the large similarities along the diagonal of the plot. Figure 8 exemplifies the similarity scores of four dummy users (Bioinformatics Scientists, Transportation Engineers, Dermatologists, and Sociology Teachers), where we observe the same behaviour when answering $Q=4$, $Q=14$, and $Q=24$ questions.

We used the Success Rate Percentage (SRP) to assess the accuracy of matching the user profile to the job roles. Briefly, this rate measures whether the correct job role occurs in the top $k$ most similar roles. More formally, the similarity $Sim\left({u}_{j}^{Q},j^{\prime}\right)$ between the dummy user ${u}_{j}^{Q}$ for the job role $j$ and all job roles $j^{\prime}\in J$ is calculated. The match is deemed successful if the true job role occurs in the top $k$ most similar roles. The $\mathrm{SRP}\left(k\right)$ is then the percentage of successful matches overall dummy users.

**Fig. 9:**
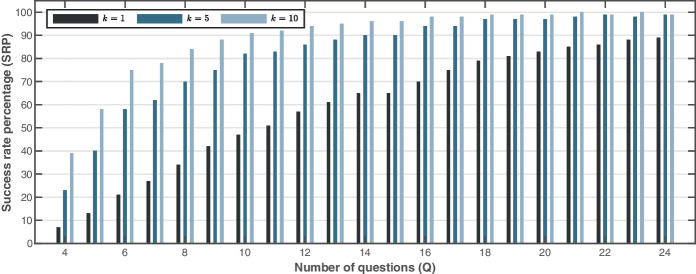
Success rate percentage on three different configurations ($k=1$, $k=5$, $k=10$) when increasing the number of questions answered by the users

Figure 9 illustrates the $\mathrm{SRP}\left(k\right)$ for $k=1$, $k=5$, and $k=10$ and dummy users constructed with $Q=\left\{4,\cdots ,24\right\}$. As expected, the success rate for matching the true job in the top 5 or 10 best matches increases rapidly with the number of questions answered. However, when the exact match $k=1$ is required, the success rate is initially proportional to $Q$, but approximately 24 questions are needed to achieve an average success rate of 90%.

**Fig. 10:**
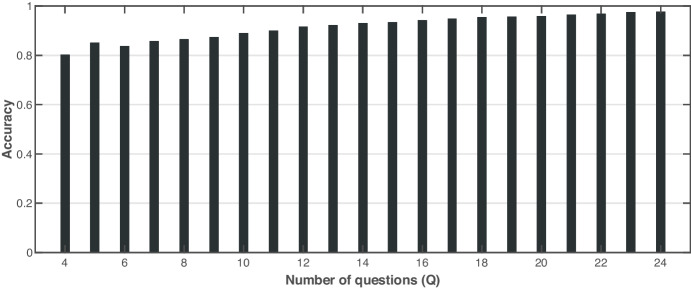
Accuracy values of the job roles assigned to the correct communities on a different number of questions

We also investigated the ability of the C3-IoC system to predict the correct job category using the reference job labels ($R$) generated by the Louvain algorithm. To compute the predicted job roles ($P$), the similarity $Sim\left({u}_{j}^{Q},j^{\prime}\right)$ between the dummy user ${u}_{j}^{Q}$ for job role $j$ and all job roles $j^{\prime}\in J$ is calculated. Then, we assign the user the job role’s reference label having the highest similarity score. The accuracy is considered the primary indicator to measure the performance of the job role matching module. Given two partitions $R$ and $P$, the accuracy measure determines the similarity between the two partitions by analysing their pairwise co-assignment of data points based on a multi-class confusion matrix. The accuracy is defined in the range $[\mathrm{0,1}]$, such that values closer to unity are preferred as they indicate a better correspondence between $R$ and $P$. Figure 10 gives information about the performance of the job role matching in terms of accuracy when increasing the number of questions answered by the user, $Q=\left\{4,\dots ,24\right\}$. In general, it is evident that the performance of the assignment of dummy user profiles to the correct communities continually increases as more questions are answered. The accuracy increases from about 80% when answering four questions to nearly 100% when the number of questions answered increases to 24 questions.

Overall, we observe that even with as few as four questions, the success rate percentage of one of the jobs in the top 10 shown to the user being appropriate to their profile is about 40%. However, when the number of questions answered increases to 24, the success rate percentage is nearly 100% accurate. This suggests that the C3-IoC tool can effectively match non-technical skills and general job roles, making a valuable contribution to user awareness and career guidance.

## User Trial Evaluation

Finally, we conducted a user trial to evaluate the C3-IoC system’s usability and usefulness. Due to the Covid-19 pandemic, the evaluation was conducted entirely online. Participants were instructed to visit and explore the C3-IoC website at their own pace.^13^ The user trial (including the survey) was designed to take around 30 min to complete and was emailed to students studying at the University of Sheffield and the University of Exeter. The survey consists of 15 questions, which are available in Appendix A. It comprises 12 closed questions on a 7-point Likert scale and three open-ended questions. The first five questions in the survey focus on evaluating the usability and usefulness of the proposed C3-IoC system. The following seven questions aim to collect demographic data. The last three questions capture information on the users’ current job or career status.

In total, we gathered 64 valid responses (N = 64) from October to December 2020. Based on the postcode address in the UK, answers indicated that 36% of participants came from Sheffield, 35% from Exeter, and 29% from other postcodes in the UK. A total of 77% of participants self-identified as male and 23% as female. The majority of the participants were aged 19–24 (72%). Also, participants were asked about their highest level of education, with “Higher education” being the majority at 80% (undergraduate and postgraduate). Regarding the field of study or area of expertise of the participants, the majority belong to the “Computer science and information technology” with 60%, followed by “Engineering, mathematics and physical sciences” and “Medicine and health” with 25% and 5% respectively.

**Fig. 11:**
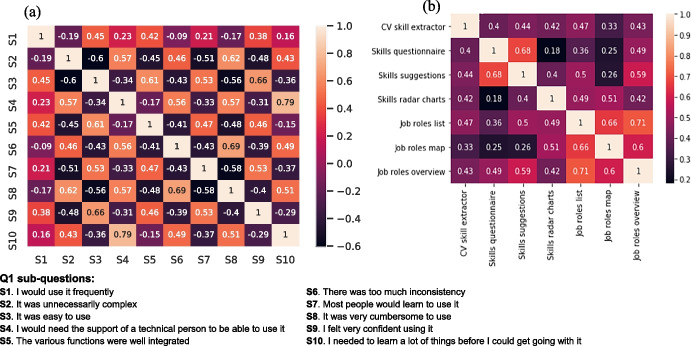
Heatmaps between statements regarding the usability of the C3-IoC system: (**a**) general usability aspects of the tool and (**b**) usability of the different C3-IoC components

**Fig. 12:**
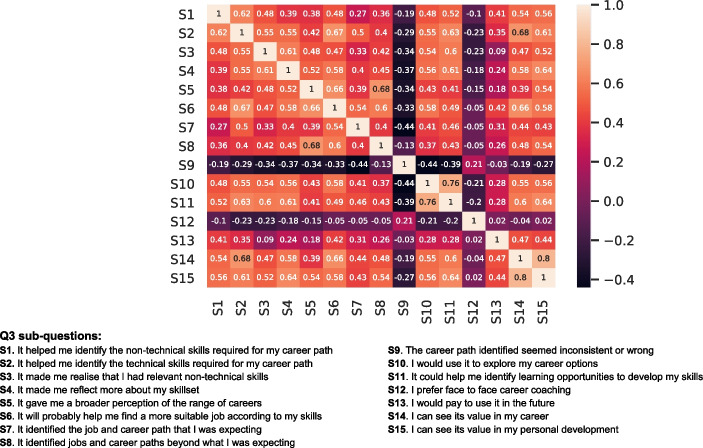
Heatmap between statements regarding the usefulness of the C3-IoC system when finding job roles based on user skills

In the following, we focus on the findings regarding evaluating the C3-IoC system in terms of its usability and usefulness corresponding to the first three questions in the survey and identified as Q1, Q2, and Q3, respectively. Figures 11 and 12 illustrate the Spearman correlation (*rs*) values between different sub-questions in Q1, Q2, and Q3. The Spearman correlation tends to the positive unit (+ 1) when observations are monotonically related and tends to the negative unit (-1) when the variables have a dissimilar rank, indicating a low association (Lehman, 2005). This is an appropriate correlation for continuous and discrete variables and less sensitive than the Pearson correlation to outliers (Dodge, 2010).

Overall, the participants provided a positive score in terms of usability of the C3-IoC system. From the 45 statement pairs in Q1, illustrated in Fig. 11a, 23 pairs showed strong correlations (*rs* > 0), indicating that the tool was easy to use, the functions were well integrated, it showed consistency, and the user felt confident using the tool. Additionally, regarding the usability of the seven components in the C3-IoC system (*CV skill extractor*, *Skills questionnaire*, *Skills suggestions*, *Skills radar charts*, *Job roles list*, *Job roles map* and *Job roles overview*) from Fig. 11b, we observe a strong relationship between all the 21 different statement pairs having all of them a positive correlation value (*rs* > 0.25). Overall, the highest-rated components were the *“Job roles map”* and the *“Job roles overview”*, which may suggest an interest in visualising or getting an overview of the job roles.

On the other hand, Fig. 12 illustrates the relationships between the Q3 sub-questions regarding the usefulness of the C3-IoC system. Among 105 pairs of statements regarding the 15 sub-questions of Q3, 61 pairs showed strong correlations (*rs* > 0.4). Specifically, there were two very strong correlations (*rs* > 0.7) between the following pairs of sub-questions: *“I would use it to explore my career options”* versus *“It could help me identify learning opportunities to develop my skill”* and *“I can see its value in my career”* versus *“I can see its value in my personal development”*. Therefore, these two pairs strongly suggest that users tend to see the tool’s value both for personal and career developments and that it made them reflect on their skillset while exploring different career options.

In general, the C3-IoC system helped users identify technical and non-technical skills in order to provide a tailored career path. Additionally, the results indicate that our proposed tool consistently provided users with both the job role and career path they were expecting and beyond their expectations, with fewer cases having experienced wrong or inaccurate results.

## Discussion and Limitations

The C3-IoC is an integrative career guidance system with unique components and features to help users explore their career paths in a rapidly changing labour market. C3-IoC comprises three modules corresponding to the (i) identification of technical and non-technical skills for existing and emerging job roles, (ii) construction of user profiles based on their personalised skills, and (iii) the creation and visualisation of job role networks (Fig. 4 and 5), and projection of user-profiles into the networks (Fig. 6). The advantages and benefits of the proposed C3-IoC system were investigated from two perspectives: its effectiveness for predicting job roles based on user profiles and its usability.

In the first experiment, the effectiveness of the similarity metric (Eq. 2) to find adequate job roles according to the user profile was performed. On the one hand, let us consider that a user profile is composed of technical and soft skills collected in three ways: answering a 24-question questionnaire (see Table 3), extracting skills from a CV, and selecting recommended skills. These skills are weighted by the user according to their experience and with the help of the system. For instance, consider the following question in the questionnaire: “*Training and Teaching: Identifying the educational skills of a teacher*”. The user could rate the question between 1 and 7, where a value close to one indicates little mastery of the skill (e.g. *give co-workers brief instructions on a simple procedural change*) and a value close to 7 indicates complete mastery of the skill (e.g. *develop and conduct training programmes for a medical school*). Thus, these tools and assistance allow for creating a robust user profile with weighted skills. On the other hand, a job role in O*NET is associated with various skills that have been weighted by their degree of importance to the corresponding job role by labour market experts. Therefore, considering the vector of skills associated with the user profile, it is possible to predict job roles in O*NET. However, as seen from the results reported in “User Trial Evaluation” section, the accuracy of job role prediction is highly dependent on the information in the user profile and, in particular, on the minimum number of questions answered from the questionnaire.

The following question was formulated: *how much information does the user need to provide to obtain job roles matching their skills?* We conducted two experiments to investigate the extent to which varying the number of questions (*q*) answered by the user. In the first experiment, the target job role is known in advance, so it is possible to measure the success rate of the proposed tool. As a result, the C3-IoC tool presents the user with the ten most similar jobs according to their skills. First, we observed that the more questions the user answers (more information about their soft skills), the more likely it is that C3-IoC will predict the appropriate job roles according to their skills (prediction of the target job role and other very similar ones). More precisely,  $k=10$ in Fig. 9 indicates that the target job role is within the group of 10 jobs generated by C3-IoC. Under this setting, it was observed that the user must answer at least six questionnaire questions to obtain a prediction percentage higher than 70%. From 10 questions onwards, a percentage equal to or higher than 90% is obtained. Therefore, it is suggested that the user answer at least six questions ($q=6$) out of 24 for C3-IoC to generate a 70% success rate in job roles (10 most similar jobs) linked to the user’s skills. On the other hand, the second experiment revealed that the C3-IoC system estimates the job community with high accuracy and, similarly to the previous experiment, as the user answers more questions, the accuracy rate is monotonically increasing (from 80 to 100% for $q=3$ and $q=24$, respectively in Fig. 10). These high accuracy values are not surprising because they are linked to job role prediction success. However, in this case, instead of predicting the job role, C3-IoC predicts the job community to which the user belongs based on their skills collected with the input questionnaire.

In the second experiment, we evaluated the usability and usefulness of the C3-IoC system and its components described in “User Trial Evaluation” section, including its capacity to recommend meaningful job roles based on the input user skills. As previously described, several users explored and tested the C3-IoC system and answered a questionnaire (see Appendix A). The users evaluated the importance and helpfulness of the different modules and components of the system (*CV skill extractor*, *Skills questionnaire*, *Skills suggestions*, *Skills radar charts*, *Job roles list*, *Job roles map* and *Job roles overview*). Additionally, the users evaluated the system’s reliability in adequately predicting job roles according to their skills, fields of study and professional interests.

Firstly, in terms of usability of the C3-IoC’s components (see Fig. 11b and Q2 in the questionnaire), the results showed that there is a strong relationship in how the following pair of components helped the users: (*Skills questionnaire, Skills suggestions*), (*Job roles list, Job roles overview*) and (*Job roles list, Job roles map*). Thus, these results suggest that based on the skill questionnaire and CV extractor, the system recommends new valuable skills to create a more complete user profile. Consequently, it provided an accurate job roles list and locations on the job map. Secondly, regarding the usefulness of the C3-IoC in finding the most appropriate job roles (see Fig. 12 and Q3 in the questionnaire with $rs>0.67$), from the evaluation results, we can highlight the following (i) the system helped the users to identify technical and soft skills required for their career path, (ii) the tool gave them a broader perspective on the range of careers as it identified jobs and career paths beyond what they were expecting, and (iii) they would use it to explore their career options as the tool could help them to identify learning opportunities to develop their skills. Therefore, the user trial evaluation revealed that they could see the C3-IoC’s value and how it could help their career and personal development.

### Limitations and Applicability

Despite the promising results of the C3-IoC tool in the two studies described previously in “Accuracy of Matching between User Profiles and Job Roles” and “User Trial Evaluation” sections, the proposed career guidance system naturally has some inherent limitations. Because it is an automatic tool based on various machine learning techniques, the quality of the results (job roles) generated by C3-IoC will depend, to a large extent, on the quality of the input information provided by the user (soft and technical skills). Therefore, the best job role generation cannot be guaranteed, and the results will show some (albeit small) variation depending on the minimum number of questions answered. The C3-IoC web application reports ten job roles ($k=10$ in Fig. 9) given a user profile that has answered a certain number of questions ($q$). Thus, we suggest the user answer at least six questions $(q\ge 6)$ out of the 24 in the questionnaire to ensure a prediction success near 75%. Indeed, the success and applicability of the tool reported in “User Trial Evaluation” section are because users mostly answered all 24 questions, obtaining a complete user profile.

## Contributions and Further Work

In this section, we highlight the main contributions of the C3-IoC system whilst signposting further areas for improvement. The C3-IoC introduces a novel knowledge-base of technical and non-technical skills relevant for careers in the IT sector that, unlike other previously mentioned career systems based on O*NET or ESCO, is built from a combination of two sources: a large corpus of job adverts and O*NET database. The job advert collection contains live information about what skills are relevant in the current labour market; thus, newly emerging job categories (e.g. *“data scientist”*) and skills (e.g. *“deep learning”*) are captured in this collection. However, the language of job adverts does not always reflect the importance or level of skill required for a certain role. Furthermore, in some adverts, essential non-technical skills such as *“teamwork”* or *“leadership”* may not be strongly emphasised or well differentiated from other non-technical skills. On the other hand, O*NET is not as reactive to changes in the job market as job adverts are, but it does attempt to provide a more thorough survey of the skills required for each role. Also, in O*NET, each skill is weighted accordingly, allowing for a more accurate picture of the job role. Although manually adapting the list of non-technical skills from the *IT job corpus* using O*NET scores and questions proved to be quite challenging and time-consuming, now that the matching process is finalised, it can be easily and automatically assigned with live information. One avenue to further implement in this regard would be the development of an ontology to ensure a common understanding of information and concepts and, the relationships between them to automate the process of accessing and querying data.

Another significant contribution of the C3-IoC system comes with the skill profiling module through the CV parser and the non-technical skills questionnaire. Both the technical skills extracted via the user’s CV and the non-technical skills questionnaire proved to be helpful for students to reflect on their skills. The CV parser can lead students to think more about writing better and more explicitly relevant CVs. Similarly, the questionnaire adapted from O*NET encourages students to self-reflect on their non-technical skills, as was confirmed in both trials (first in the pilot study and second in the evaluation of the latest version of the C3-IoC). For Computer Science graduates, whose unemployment rates remain high, this self-assessment and identification of skills can be a valuable exercise in itself by raising awareness. Students can also see how improving or developing certain skills moves them closer to their target job role. Nonetheless, as it is currently implemented, the CV parser cannot recognise skills described in complex ways, for instance inferring *“leadership”* from a sentence like *“I led a team of 14…”*, nor can it infer level, such as differentiating basic ability in programming Python from expert-level ability. Improvements in the CV parser could help characterise and locate the user more accurately. The questionnaire adapted from O*NET has been extensively used, but its wording, particularly in the examples, is inherently related to the North American context, e.g. *“high school”*. This issue was also reported in some of the comments made by the students of the second trial. Therefore, those examples can mislead and mean different things to other cultures and require proper adaptation to maintain their sense without losing their research-based validity. Replacing some examples with local and IT-specific expressions could tailor the questionnaire to students’ backgrounds and experiences.

Finally, by associating different jobs with various technical and non-technical skills, we can project any individual in the job landscape, identifying similarities between jobs and matching their skills with a certain role. In both the preliminary and second trials, students have identified the network visualisation as a helpful way to explore and plan their careers. Nonetheless, to better cope with emerging job roles and skills, we acknowledge the need to refine the set of machine learning techniques used in our proposed approach that could be improved. For example: (a) research different network projections to find a better relationship between jobs roles with similar skills; (b) explore alternative techniques for the identification of network communities on the resulting job roles landscapes, for instance, methods based on data clustering; (c) try different similarity metrics that can provide more accurate matchings between user-profiles and job roles (d) explore, apply and extend the proposed approach to use constantly up-to-date databases, e.g., from *“live”* job adverts.

## Conclusion

In this paper we have described an AI-based solution named C3-IoC that allows students to visualise and explore the job role space according to their skillset. The principal components of the C3-IoC system are: (*Module 1*) a framework involving a hybrid model based on the *IT job corpus* and the *general job corpus*; (*Module 2*) profiling of technical and non-technical skills; and (*Module 3*) a visualisation and matching process to find the most suitable job role for a set of skills.

The proposed C3-IoC system was evaluated in two ways: (i) in a methodological way, where the accuracy of the tool in predicting work roles from different user profiles was measured quantitatively, and (ii) in a didactic way, where different users (students) evaluated the usability and usefulness of the various components of the C3-IoC system. These studies showed that the proposed tool could offer assistance in preparing students for the workforce and be useful for higher education career guidance services.

We believe our proposed approach can provide effective insights in a market full of uncertainty and unpredictability (Ponce del Castillo, 2018) impacted by both Covid 19 and rapidly emerging AI. Despite building on student CVs and job vacancies extracted from the IT sector in the UK, the scope of application of the C3-IoC system can be enlarged. Indeed, the exploration of career options is increasingly done by people at all life stages, not only by students in school-to-work transitions. Equally important in future developments of the C3-IoC system is to provide tailored guidance about education and training opportunities, forwarding users to courses that focus on skills relevant to their target job roles. The system could complement career guidance in schools and universities, helping counsellors stay abreast of the rapidly-evolving job market and help navigate the nuances of traversing areas between IT and non-IT sectors.

## Supplementary Information

Below is the link to the electronic supplementary material.

Supplementary file1 (PDF 1.13 MB)

## Footnotes

1 https://www.c3-ioc.co.uk/

2 https://www.careeronestop.org/

3 https://www.focus2career.com/

4 https://www.mynextmove.org/

5 https://www.morrisby.com/

6 https://www.prospects.ac.uk/

7 https://www.myersbriggs.org/my-mbti-personality-type/mbti-basics/

8 https://www.themyersbriggs.com/en-US/

9 https://careerscope.uk.net/

10 https://www.careeronestop.org/

11 https://findajob.dwp.gov.uk/

12 https://www.onetonline.org/

13 Once they reached the end of the session, they were prompted with the option to complete a short survey about their experience and were given a £5 voucher for participating in the trial.
