# Supplementary material for: C3-IoC: A Career Guidance System for Assessing Student Skills using Machine Learning and Network Visualisation
Source: Int J Artif Intell Educ. 2022 Dec 1:1–28. Online ahead of print. doi: 10.1007/s40593-022-00317-y (PMC9715283; doi:10.1007/s40593-022-00317-y)
Supplement: Supplementary file 1 — Supplementary file1 (PDF 1.13 MB) [file 40593_2022_317_MOESM1_ESM.pdf]

Appendix A: User trial evaluation questionnaire

<table>
<tr><td>Question 1 (Q1)</td><td>Please indicate your level of agreement with the following statements regarding the usability of the C^3 - IoC:</td></tr>
<tr><td>Answer format</td><td><table><tr><td>1
(Strongly disagree)</td><td>2</td><td>3</td><td>4
(Neither disagree nor agree)</td><td>5</td><td>6</td><td>7
(Strongly agree)</td></tr></table></td></tr>
<tr><td>Sub-question</td><td>I would use it frequently</td></tr>
<tr><td>Sub-question</td><td>It was unnecessarily complex</td></tr>
<tr><td>Sub-question</td><td>It was easy to use</td></tr>
<tr><td>Sub-question</td><td>I would need the support of a technical person to be able to use it</td></tr>
<tr><td>Sub-question</td><td>The various functions were well integrated</td></tr>
<tr><td>Sub-question</td><td>There was too much inconsistency</td></tr>
<tr><td>Sub-question</td><td>Most people would learn to use it</td></tr>
<tr><td>Sub-question</td><td>It was very cumbersome to use</td></tr>
<tr><td>Sub-question</td><td>I felt very confident using it</td></tr>
<tr><td>Sub-question</td><td>I needed to learn a lot of things before I could get going with it</td></tr>
<tr><td>Question 2 (Q2)</td><td>In terms of usefulness, how would you rate the following C^3 - IoC components?</td></tr>
<tr><td>Answer format</td><td><table><tr><td>1
(Not at all useful)</td><td>2</td><td>3</td><td>4
(Neutral)</td><td>5</td><td>6</td><td>7
(Extremely useful)</td></tr></table></td></tr>
<tr><td>Sub-question</td><td>CV skill extractor</td></tr>
<tr><td>Sub-question</td><td>Skills questionnaire</td></tr>
<tr><td>Sub-question</td><td>Skills suggestions</td></tr>
<tr><td>Sub-question</td><td>Skills radar charts</td></tr>
<tr><td>Sub-question</td><td>Job roles list</td></tr>
<tr><td>Sub-question</td><td>Job roles map</td></tr>
<tr><td>Sub-question</td><td>Job roles overview</td></tr>
</table>

<table>
<tr><td>Question 3 (Q3)</td><td>Please indicate your level of agreement with the following statements regarding the usefulness of C^3 - IoC:</td></tr>
<tr><td>Answer format</td><td><table><tr><td>1
(Strongly disagree)</td><td>2</td><td>3</td><td>4
(Neither disagree nor agree)</td><td>5</td><td>6</td><td>7
(Strongly agree)</td></tr></table></td></tr>
<tr><td>Sub-question</td><td>It helped me identify the non-technical skills required for my career path</td></tr>
<tr><td>Sub-question</td><td>It helped me identify the technical skills required for my career path</td></tr>
<tr><td>Sub-question</td><td>It made me realise that I had relevant non-technical skills</td></tr>
<tr><td>Sub-question</td><td>It made me reflect more about my skillset</td></tr>
<tr><td>Sub-question</td><td>It gave me a broader perception of the range of careers</td></tr>
<tr><td>Sub-question</td><td>It will probably help me find a more suitable job according to my skills</td></tr>
<tr><td>Sub-question</td><td>It identified the job and career path that I was expecting</td></tr>
<tr><td>Sub-question</td><td>It identified jobs and career paths beyond what I was expecting</td></tr>
<tr><td>Sub-question</td><td>The career path identified seemed inconsistent or wrong</td></tr>
<tr><td>Sub-question</td><td>I would use it to explore my career options</td></tr>
<tr><td>Sub-question</td><td>It could help me identify learning opportunities to develop my skills</td></tr>
<tr><td>Sub-question</td><td>I prefer face to face career coaching</td></tr>
<tr><td>Sub-question</td><td>I would pay to use it in the future</td></tr>
<tr><td>Sub-question</td><td>I can see its value in my career</td></tr>
<tr><td>Sub-question</td><td>I can see its value in my personal development</td></tr>
<tr><td>Question 4 (Q4)</td><td>To what extent did you find the C^3 website to be:</td></tr>
<tr><td>Answer format</td><td><table><tr><td>1
(Not at all)</td><td>2</td><td>3</td><td>4
(Neutral)</td><td>5</td><td>6</td><td>7
(Extremely)</td></tr></table></td></tr>
<tr><td>Sub-question</td><td>Inclusive</td></tr>
<tr><td>Sub-question</td><td>Accessible</td></tr>
<tr><td>Question 5 (Q5)</td><td>Do you have any additional comments about the C^3 - IoC?</td></tr>
<tr><td>Answer format</td><td>Long free text</td></tr>
</table>

**Question 6 (Q6)**	**Age:**
	16-18 / 19-24 / 25-40 / 41-54 / 55-64 / 65 and over / I do not want to declare
**Question 7 (Q7)**	**Gender identity:**
Answer format	Male / Female / Non-binary / I do not want to declare
**Question 8 (Q8)**	**Please choose one option that best describes your ethnic group or background:**
Answer format	White - English/Welsh/Scottish/Northern Irish/British<br>White - Irish<br>White - Gypsy or Irish Traveller<br>Other White background<br>Mixed/Multiple - White and Black Caribbean<br>Mixed/Multiple - White and Black African<br>Mixed/Multiple - White and Asian<br>Other Mixed/Multiple ethnic background<br>Asian/Asian British - Indian<br>Asian/Asian British - Pakistani<br>Asian/Asian British - Bangladeshi<br>Asian/Asian British - Chinese<br>Other Asian background<br>Black/African/Caribbean - African<br>Black/African/Caribbean - Caribbean<br>Other Black/African/Caribbean background<br>Arab<br>Other ethnic group<br>I do not want to declare

**Question 9 (Q9)**	**Please indicate the first letters of your postcode address in the UK:**
Answer format	Short free text
**Question 10 (Q10)**	**Please state if you have any of the following disabilities or conditions:**
Answer format	Yes / No
Sub-question	No known disability
Sub-question	I do not want to declare
Sub-question	Two or more impairments and/or disabling medical conditions
Sub-question	Specific learning difficulty such as dyslexia, dyspraxia and AD(H)DA
Sub-question	Social/communication impairment such as Asperger's syndrome/other autistic spectrum disorder
Sub-question	Long standing illness or health condition such as cancer, HIV, diabetes, chronic heart disease or epilepsy
Sub-question	Mental health condition, such as depression, schizophrenia or anxiety disorder
Sub-question	Physical impairment or mobility issues, such as difficulty using arms or using a wheelchair or crutches
Sub-question	Deaf or a serious hearing impairment
Sub-question	Blind or a serious visual impairment uncorrected by glasses
Sub-question	A disability, impairment or medical condition that is not listed above
**Question 11 (Q11)**	**Please state your highest level of education:**
Answer format	Other / Elementary education / Secondary education / Higher education - undergraduate / Higher education - postgraduate
**Question 12 (Q12)**	**Please select your field of study or area of expertise:**
Answer format	Other / Computer Science and Information Technology / Engineering, Mathematics and Physical Sciences / Arts and Humanities

	Life and Environmental Sciences<br>Social Sciences<br>Medicine and Health

**Question 13 (Q13)**	**What is your current employment status?**
Answer format	Employed full-time | Employed part-time | Retired | Student | Unemployed/Not in paid work
**Question 14 (Q14)**	**Please indicate your job title.**
Answer format	Short free text
**Question 15 (Q15)**	**If you have any particular job or career in mind, can you tell us what is it?**
Answer format	Short free text

Appendix B: Demonstrative example of the C3-IoC system

In the following, we present a demonstrative example of the proposed C3-IoC system in three steps when trying to target a Database Manager Profile.

Step I. Skill Evaluation

Let us suppose that the trial user responds to the first six questions of the input questionnaire (see Fig. 1 in the Appendix). Then, for didactic proposes, the confidence level selected is 4 (levels: NA, 1,2,3,4,5,6,7 such that the higher the value, the best is the confidence level of the user):
(1) Attention to Detail. Being focused, attentive to details and thorough in completing tasks: **4**
(2) Management. Getting members of a group to work together to accomplish tasks and determining how money will be spent to get the work done: **4**
(3) Self-Control. Maintaining composure, keeping emotions in check and dealing effectively with high-stress situations: **4**
(4) Mathematical Reasoning. Choosing the right mathematical methods or formulas to solve a problem: **4**
(5) Training and Teaching. Identifying the educational needs of others, providing guidance and training to help others to improve their knowledge or skills**: 4**
(6) Mathematical Reasoning. Choosing the right mathematical methods or formulas to solve a problem**: 4**

Fig. 1 Example of answering the *Attention to Detail* question in the input questionnaire.

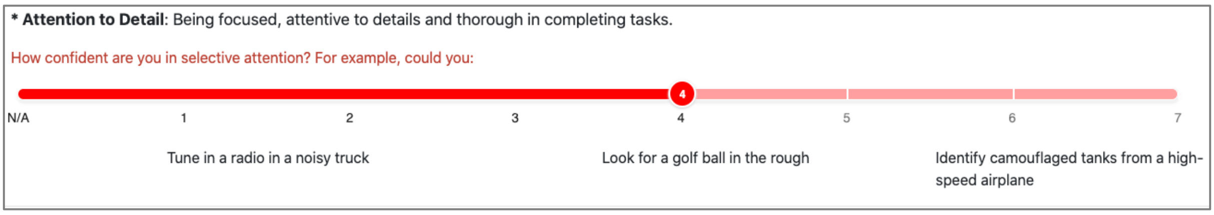

Step 2. Skill Profile

Let us select skills related to the target profile (see Fig. 2 in the Appendix). These soft and technical skills complement the ones selected in Step 1. For example, some soft skills added manually are Interacting with computers, Processing Information, and Database Administration, whereas some technical skills are Relational Databases, SQL, and PostgreSQL.

Fig. 2 List of soft and technical skills selected according to the target Database Manager Profile.

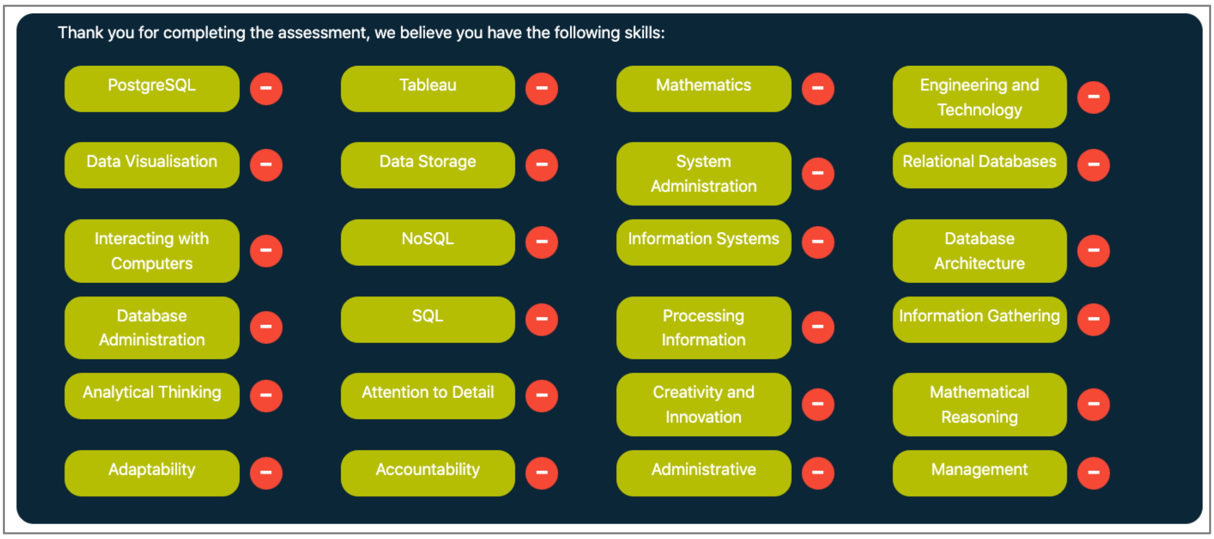

Following with Skill Profile stage, in Fig 3, we can observe some of the technical skills grouped into four categories: Programming Language, Tools and Platforms, General Tech and Specific Training. Also, Fig 4 illustrates some soft skills categorised in Social, Thinking, Personal and Management. In this stage, the user can choose a confidence level for each skill, and the radar chart will be updated accordingly.

Fig. 3 Illustration of the technical skills selected for the target Database Manager Profile.

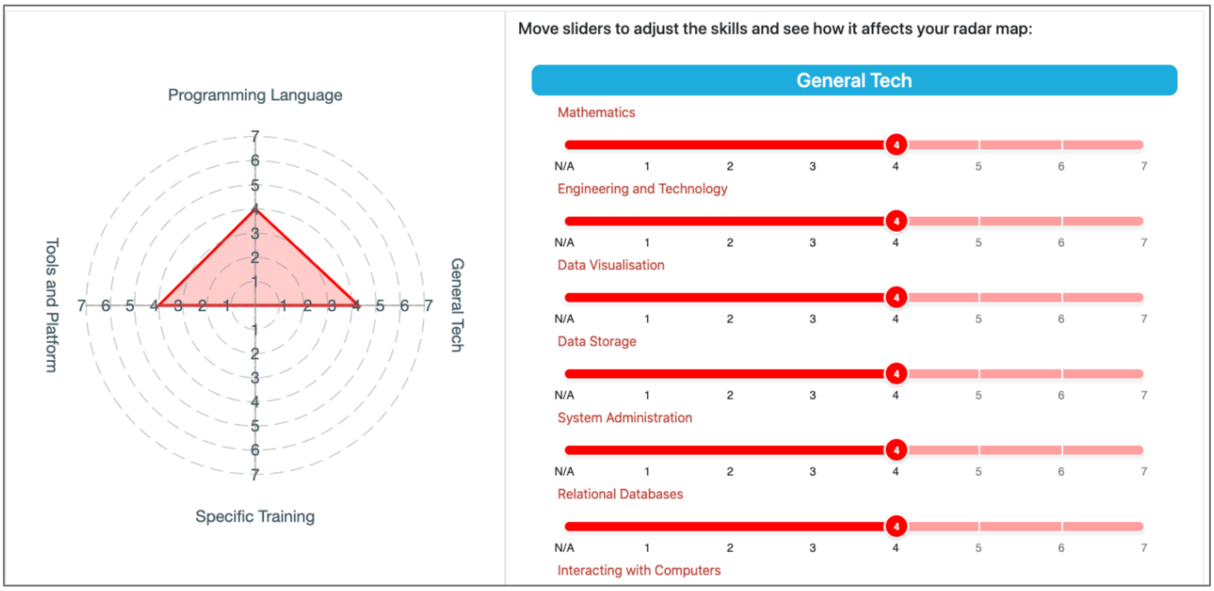

Fig. 4 Illustration of the soft skills selected for the target Database Manager Profile.

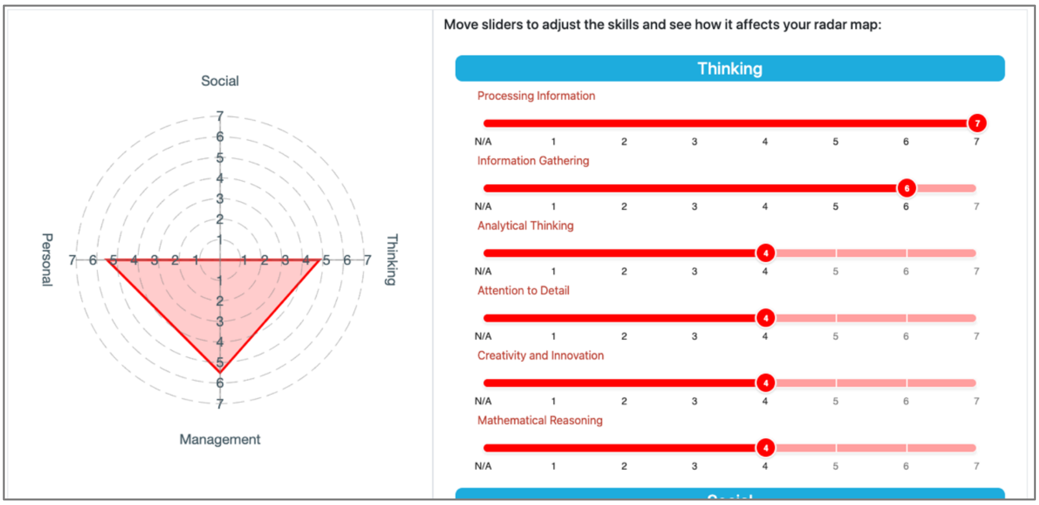

Step 3. Job Role Explorer

On the one hand, if we focus on the IT job role map in Fig. 5, it is evident that the job role obtained is "Database Administrator" and that there are other very similar roles such as "System Analysis" and "Data Analysis". However, on the other hand, note that the user does not have other essential technical skills for this position.

Fig. 5 Illustration of IT job roles obtained based on the input user profile.

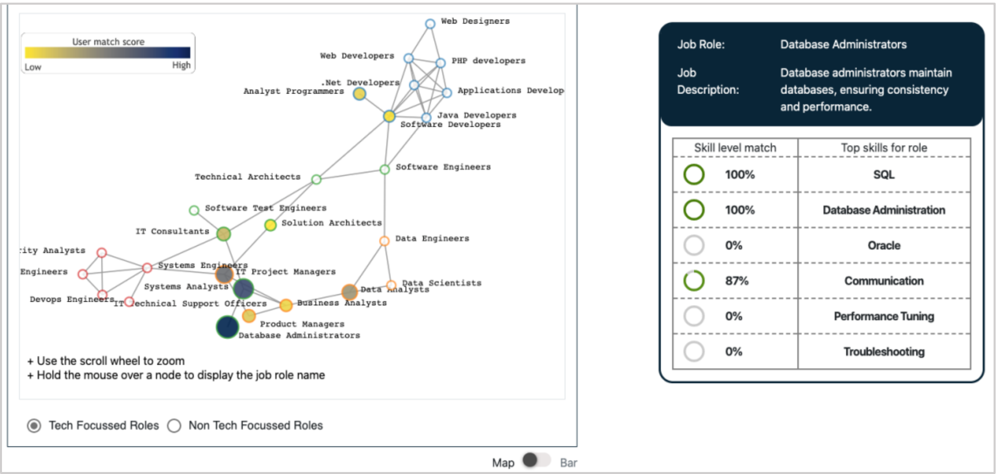

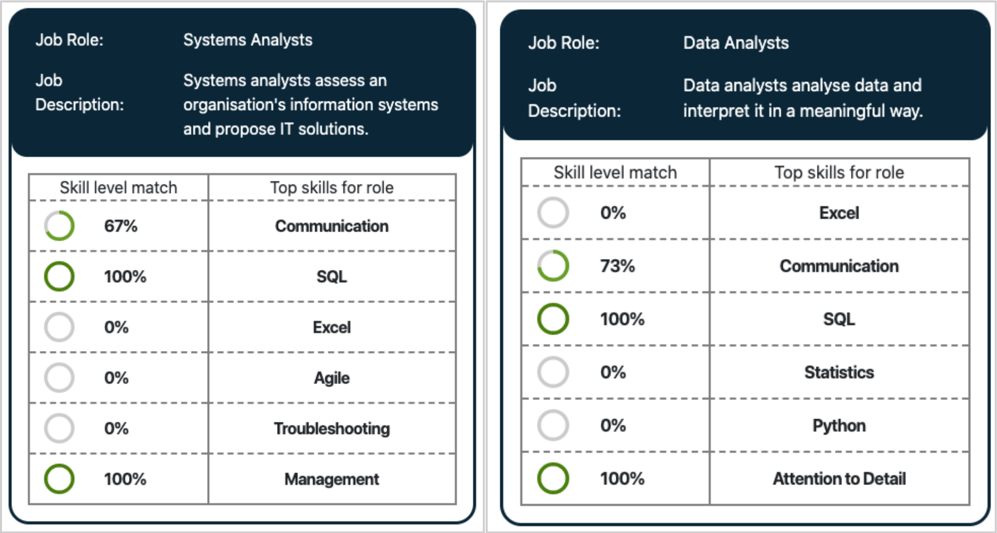

Finally, when we look at the results obtained for O*NET job roles based on soft skills from Fig. 6, we observe that one of the recommended job roles is “Data Administrators” and that other similar job roles are “Computer Programmers”, “Survey Researchers”, and “Statistical Assistants”. Concerning the soft features, we can observe how a set of features are transversal to the mentioned job roles. In addition, the system indicates to the user the skills required for the different job roles.

Fig. 6 Illustration of O*NET job roles obtained based on the input user profile.

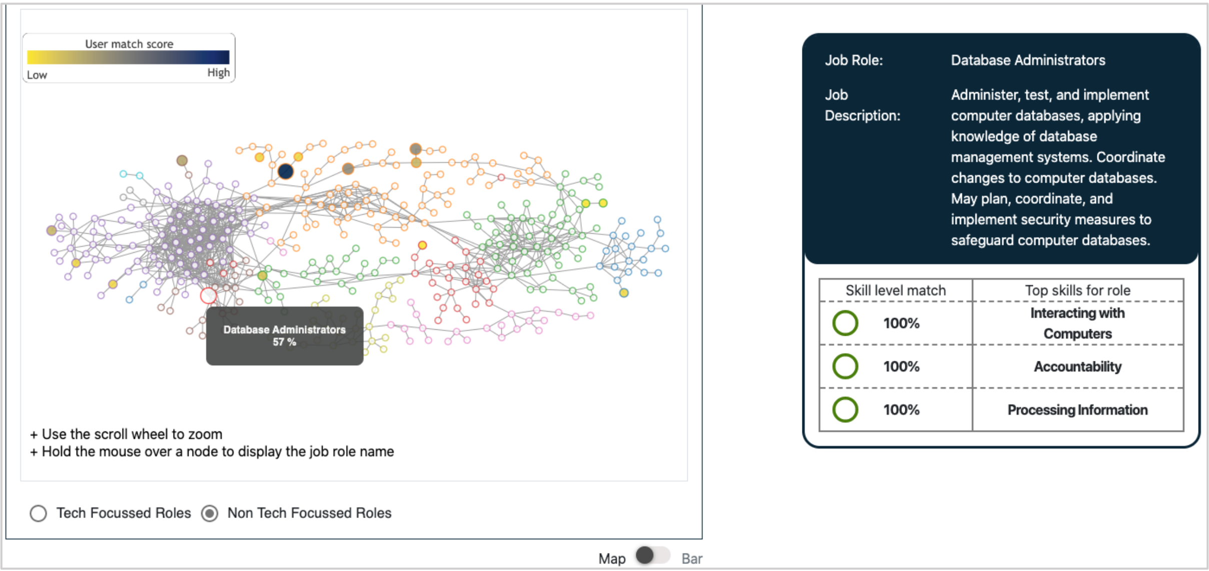

Job Role: Computer Programmers

Job Description: Create, modify, and test the code, forms, and script that allow computer applications to run. Work from specifications drawn up by software developers or other individuals. May assist software developers by analyzing user needs and designing software solutions. May develop and write computer programs to store, locate, and retrieve specific documents, data, and information.

Skill level match	Top skills for role
100%	**Interacting with Computers**
0%	**Programming**
100%	**Accountability**
100%	**Processing Information**

Job Role: Survey Researchers

Job Description: Plan, develop, or conduct surveys. May analyze and interpret the meaning of survey data, determine survey objectives, or suggest or test question wording. Includes social scientists who primarily design questionnaires or supervise survey teams.

Skill level match	Top skills for role
100%	**Interacting with Computers**
100%	**Accountability**
63%	**Analytical Thinking**
94%	**Information Gathering**
100%	**Processing Information**

Job Role: Statistical Assistants

Job Description: Compile and compute data according to statistical formulas for use in statistical studies. May perform actuarial computations and compile charts and graphs for use by actuaries. Includes actuarial clerks.

Skill level match	Top skills for role
100%	**Interacting with Computers**
65%	**Mathematical Reasoning**
97%	**Information Gathering**
0%	**Dependability**
68%	**Analytical Thinking**
